# Detection of the dominant pathogens in diarrheal calves of Ningxia, China in 2021–2022

**DOI:** 10.3389/fvets.2023.1155061

**Published:** 2023-04-17

**Authors:** Dong Wang, Haihui Gao, Long Zhao, Changrong Lv, Wei Dou, Xiuping Zhang, Yong Liu, Xiaodong Kang, Kangkang Guo

**Affiliations:** ^1^College of Veterinary Medicine, Northwest A&F University, Xianyang, Shaanxi, China; ^2^Institute of Animal Science, Ningxia Academy of Agriculture and Forestry Sciences, Yinchuan, China

**Keywords:** diarrhea, calf, epidemic investigation, Ningxia, pathogens

## Abstract

**Introduction:**

Calf diarrhea is a complex disease that has long been an unsolved problem in the cattle industry. Ningxia is at the forefront of China in the scale of cattle breeding, and calf diarrhea gravely restricts the development of Ningxia's cattle industry.

**Methods:**

From July 2021 to May 2022, we collected diarrhea stool samples from calves aged 1–103 days from 23 farms in five cities in Ningxia, and performed PCR using specific primers for 15 major reported pathogens of calf diarrhea, including bacteria, viruses, and parasites. The effect of different seasons on the occurrence of diarrhea in calves was explored, the respective epidemic pathogens in different seasons were screened, and more detailed epidemiological investigations were carried out in Yinchuan and Wuzhong. In addition, we analyzed the relationship between different ages, river distributions and pathogen prevalence.

**Results:**

Eventually, 10 pathogens were detected, of which 9 pathogens were pathogenic and 1 pathogen was non-pathogenic. The pathogens with the highest detection rate were *Cryptosporidium* (50.46%), Bovine rotavirus (BRV) (23.18%), *Escherichia coli* (*E. coli*) K99 (20.00%), and Bovine coronavirus (BCoV) (11.82%). The remaining pathogens such as Coccidia (6.90%), Bovine Astrovirus (BoAstV) (5.46%), Bovine Torovirus (BToV) (4.09%), and Bovine Kobuvirus (BKoV) (3.18%) primarily existed in the form of mixed infection.

**Discussion:**

The analysis showed that different cities in Ningxia have different pathogens responsible for diarrhea, with *Cryptosporidium* and BRV being the most important pathogens responsible for diarrhea in calves in all cities. Control measures against those pathogens should be enforced to effectively prevent diarrhea in calves in China.

## Introduction

Diarrhea is one of the most important diseases that damages the health of calves worldwide. It is considered to be one of the diseases causing the highest economic losses to the cattle industry, with losses of up to 10 million dollars due to calf diarrhea in Norway in 2006, followed by cases of varying degrees of calf diarrhea reported in the United States in 2007, South Korea in 2013, and Pakistan in 2014 (1, 2). The main causes of calf diarrhea are intricate and complex (3, 4). In addition to genetics, age, herd and farm environment, feeding practices, poor management and other complications, the most important factor is infection (5, 6). Many countries, including China, have experienced calf diarrhea outbreaks of differing degrees caused by pathogens, such as *Cryptosporidium*, BRV, BCoV, *E. coli* K99 and other pathogens (7–10). According to the annual report of Japan in 2017, the economic losses caused by BRV in the previous years were estimated to be about 1 billion yen (11). In addition to causing diarrhea, *Cryptosporidium*, BCoV, and *E.coli* K99 also have different effects on increasing mortality, reducing immunity, and reducing milk production (12, 13).

In China, calf diarrhea outbreaks have been reported in many provinces and regions (14–17), but Ningxia has few reports in the article that has comprehensively and systematically investigated the epidemic situation and pathogen distribution characteristics of calf diarrhea. Ningxia has a natural and favorable breeding environment, coupled with the government policy support for the cattle breeding industry, making it one of the important cattle breeding areas in China. With the growing scale of the cattle industry in Ningxia, diarrhea in calves has become an increasingly serious problem, such as the absence of clinical symptoms in calves carrying the pathogen, the rapid spread of the pathogen, and the effect of different environments on the occurrence of diarrhea, which have not been reported or studied.

In order to investigate the prevalence of calf diarrhea in Ningxia and clarify the main pathogens that cause calf diarrhea prevalence in different cities, and study the effects of different seasons to diarrhea in calves, calf diarrhea fecal samples were collected from 23 large-scale cattle farms in five cities of Yinchuan, Wuzhong, Shizuishan, Zhongwei and Guyuan. Pathogens that have been reported to be associated with calf diarrhea were tested, including *E. coli* K99 (18), *Salmonella* (19), *Proteus mirabilis* (20), *Clostridium perfringens* (*C. perfringens*) (21), Bovine Viral Diarrhea Virus (BVDV) (22), BRV (23), BCoV (23), BToV (22), BoAstV (24), BKoV (24), Bovine Norovirus (BNoV) (24), Bovine Enterovirus (BEV) (25), *Cryptosporidium* (26), *Coccidia* (27, 28), and *Giardia* (29). The prevalence and distribution characteristics of these pathogens were analyzed to develop a reasonable and effective treatment plan for diarrhea in calves and to provide basic data for the prevention of diarrhea in calves.

## Materials and methods

### Sampling

**Figure 1:**
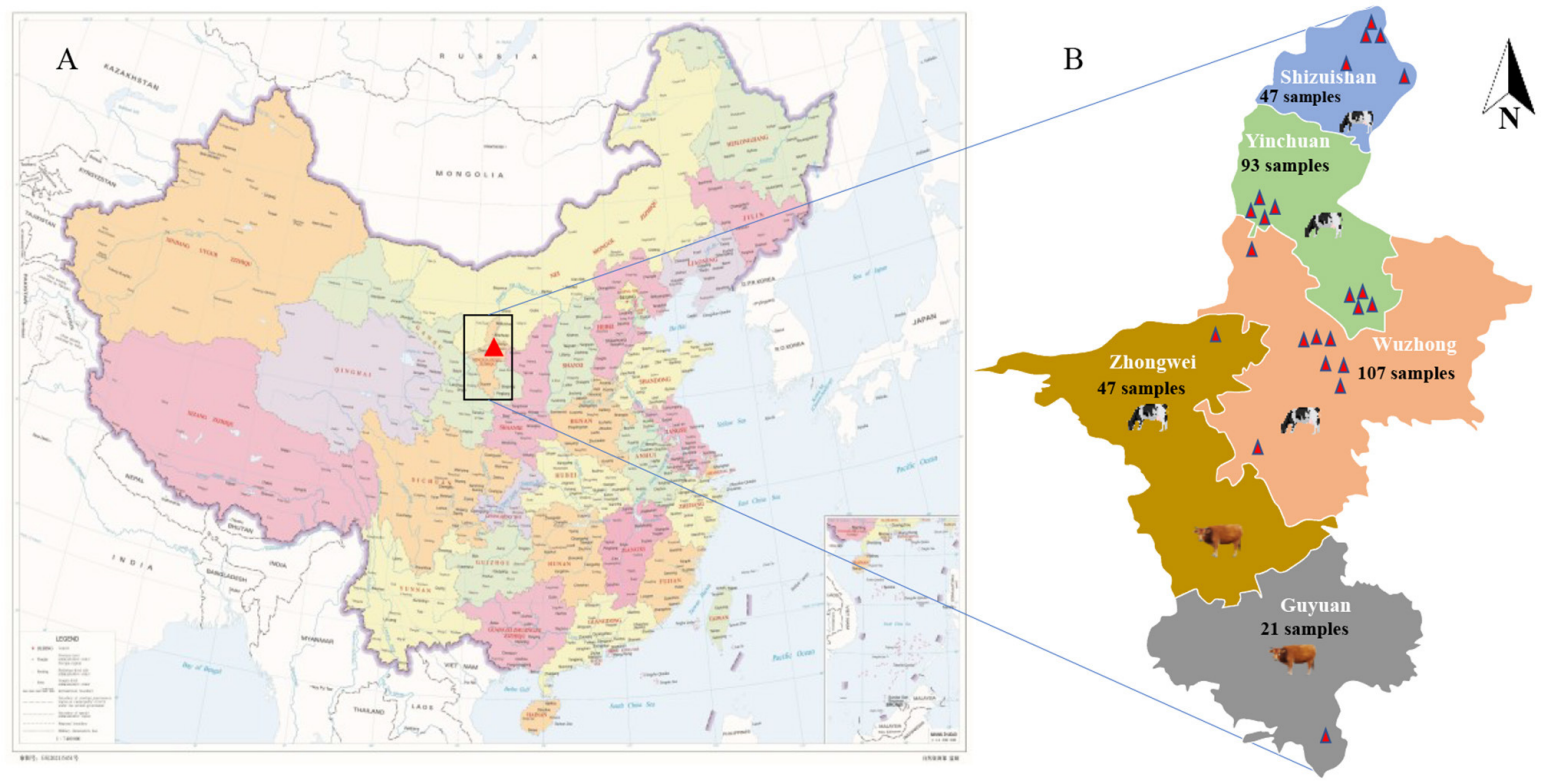
Cattle farm location and sampling information. **(A)** The geographical location of Ningxia Hui Autonomous Region (red marked as Ningxia Hui Autonomous Region, red star marked as Beijing, the capital of China). **(B)** The geographical location of the cattle farm and the total number of samples collected in each city (different colors represent different cities).

From July 2021 to May 2022, 315 calf stool samples including 220 fresh calf stool samples with diarrhea and 95 fresh normal samples from 23 large-scale cattle farms in 5 cities of Ningxia were collected. Using sterile disposable gloves to collect normal calf rectal stool samples; 4 mL fetal calf serum(FBS)-free DMEM was taken to a sterile 15 mL tube, and the diarrhea stool samples were collected into the tube and stored at 4°C. The common symptoms of diarrheal calves were dehydration, loss of appetite, watery diarrhea, and mental depression. Figure 1A shows the geographical location of the Ningxia Hui Autonomous Region, and Figure 1B shows the geographical location of the cattle farm and the total number of samples collected in each area. Table 1 shows the specific sampling numbers in Ningxia.

**Table 1:** The total number of samples with diarrhea and non-diarrhea in five cities.

**Location**	**Number of the calves of diarrhea**	**Number of non-diarrhea calves**
Yinchuan	68	25
Wuzhong	70	37
Shizuishan	35	12
Zhongwei	38	9
Guyuan	9	12
Total	220	95

### DNA extraction

After the collected fresh stool was transported back to the laboratory at 4°C, 200 g of stool were dispensed into 2 mL sterile EP tubes on a sterile clean bench, and total DNA was extracted using stool DNA kit (OMEGA, Georgia, USA), and then PCR detection was performed to detect *E. coli* K99, *Salmonella, Proteus mirabilis, C. perfringens, Coccidia, Cryptosporidium, Giardia*.

### RNA extraction and reverse transcription

The collected fresh diarrhea stool samples were diluted with 0.9% sterile normal saline. After repeated freezing and thawing at −80°C for three times, the samples were centrifuged at 4°C, 12,000 r/min for 5 min, and the supernatant was collected. Total RNA was extracted from stool using Trizol reagent *AG RNA ex Pro* (Accurate Biotechnology, Hunan, China). According to the manufacturer's operating rules, 2 μg total RNA was reverse transcribed into cDNA using *Evo M-MLV* RT Mix kit with gDNase. The cDNA was used to detect viruses that caused bovine diarrhea such as BVDV, BRV, BCoV, BToV, BoAstV, BKoV, BNoV, and BEV.

### Identification and detection of pathogens by PCR

The primers used to detect the above pathogens are shown in Table 2. The extracted RNA was measured using NanoDrop One (Thermo Fisher Scientific, Waltham, MA, USA) and the RNA concentration was in the normal range. Each sample was taken 2 μg RNA for reverse transcription to obtain the same concentration of cDNA. Nested PCR was performed to detect *Cryptosporidium* and *Giardia* using 2 × Taq Master Mix (Vazyme Biotech, Nanjing, China), the specific PCR system was 2 × Taq Master mix 10 μL, upstream and downstream primers 1 μL, template 2 μL, supplemented with ddH_2_O to 20 μL, the primer concentration was 10 μM. PCR amplification of other pathogens was carried out using 2 × M5 HiPer plus Taq HiFi PCR mix (Mei5 biotechnology, Beijing, China), the specific PCR system was 2 × M5 HiPer plus Taq HiFi PCR mix 10 μL, upstream and downstream primers 1 μL, template 2 μL, supplemented with ddH_2_O to 20 μL, the primer concentration was 10 μM.

**Table 2:** Primers used for PCR.

**Pathogens species**	**Primer**	**Sequence (5^′^-3^′^)**	**Product size (bp)**	**References**
*E. coli* K99	*F5*	F: TATTATCTTAGGTGGTATGG	314	(18)
		R: GGTATCCTTTAGCAGCAGTATTTC		
BCoV	Nsp10 of ORF1a	F: CGAGTTGAACACCCAGAT	230	(23)
		R: GAGACGGGCATCTACACT		
BRV	VP6	F: CCACCAGGTATGAATTGGAC	231	
		R: GAGTAATCACTCAGATGGCG		
BNoV	RdRp	F: AGTTAYTTTTCCTTYTAYGGBGA	532	(20)
		R: AGTGTCTCTGTCAGTCATCTTCAT		
BKoV	3D	F: TGGAYTACAAGRATGTTTTGATGC	216	
		R: TGTTGTTRATGATGGTGTTGA		
BoAstV	ORF1a	F: GAYTGGACBCGHTWTGATGG	432	
		R: KYTTRACCCACATNCCAA		
BEV	5′-UTR	F: AGCAACACTGGATTGTGCG	416	(25)
		R: GGAGTAGTCCGACTCCGC		
BVDV	5′-UTR	F: GCTAGCCATGCCCTTAG	290	(22)
		R: CCATGTGCCATGTACAG		
BToV	M	F: TTCTTACTACACTTTTTGGA	603	
		R: ACTCAAACTTAACACTAG AC		
*Cryptosporidium*	18S rRNA F1	F: TTCTAGAGCTAATACATGCG	1325	(6)
	18S rRNA R1	R: CCCATTTCCTTCGAAACAGGA		
	18S rRNAF2	F: GGAAGGGTTGTATTTATTAGATAAAG	830	
	18S rRNA R2	R: AAGGAGTAAGGAACAACCTCCA		
*Giardia*	TPI AL3543	F: AAATIATGCCTGCTCGTCG	605	(30)
	TPI AL3546	R: CAAACCTTITCCGCAAACC		
	TPI AL3544	F: CCCTTCATCGGIGGTAACTT	530	
	TPI AL3545	R: GTGGCCACCACICCCGTGCC		

### Detection *Coccidia*

Take 2 g stools sample of diarrheal calves (≥18 d), put it into a beaker, add 5 mL of water first, stir and mix well, add saturated saline to 60 mL, filter through a copper mesh after mixing, absorb the stool liquid, and inject it into McMaster Egg Slide Counting Chamber, after stewing for 5 min, count the number of EPG (Egg Per Gram) or OPG (Oocysts Per Gram) in the two graduated chambers under the microscope (27, 28).

The average A of the number of eggs in the two counting chambers multiplied by 200 is the number of eggs or oocysts per gram of stool. Compute the amount of EPG or OPG of oocysts per gram of stool according to the following formula:


$$\begin{array}{l} \text{EPG}/\text{OPG }=\;\left[\left(\text{n}1\;+\text{ n}2\right)/\left(2\times 0.15\right)\right]\times 60÷2\;=\text{ A}\times \;200 \end{array}$$

### Statistical analysis

All PCR products were visualized on a 1.0% agarose gel. All positive samples were purified and sequenced by Tsingke Biotechnology (Beijing, China). The sequence results were aligned in GenBank.

The correlation between the pathogen detection rate and the distance between the cattle farm and the river was analyzed using GraphPad, version 9.0.0. Statistical analyses of pathogen detection rates in different seasons throughout Ningxia and in different seasons in Yinchuan and Wuzhong were performed using GraphPad, version 9.0.0. Chi-square tests were performed at a 5% level of significance in SPSS 20.

## Results

### Detection of different pathogens by PCR

#### Cryptosporidium

PCR detection using primers designed by Xiao et al. (6), 111 (50.46%) of 220 stool samples were positive, of which 53 (24.09%) were infected by *Cryptosporidium* alone, and the rest were mixed infection (26.36%). The two highest proportions of mixed infections were *Cryptosporidium* and *E. coli* K99 (5.91%), followed by *Cryptosporidium* and BRV (5.45%), and then *Cryptosporidium* and Giardia (4.09%).

#### Giardia

PCR detection using primers designed by Sulaiman (30), 30 (13.64%) of 220 stool samples were positive, of which 9 (4.09%) were infected by *Giardia* alone and the rest were mixed infection (9.55%). The two highest proportions of mixed infections were *Giardia* and *Cryptosporidium* (4.09%), followed by *Giardia* & *Cryptosporidium* & *E. coli* K99, *Giardia* & *Cryptosporidium* & BRV, *Giardia* & *Cryptosporidium* & BCoV, with a detection rate of 0.91%.

#### *E. coli* K99

PCR detection using the primers reported by Keykhaei (18), among the 220 stool samples, 44 (20.00%) were positive, of which 14 (6.36%) were infected by *E. coli* K99 alone, and the rest were mixed infection (13.64%). The two highest proportions of mixed infections are *E. coli* K99 and *Cryptosporidium* (7.73%), followed by *E. coli* K99 and BRV (4.09%), and the proportion of simultaneous infection of *E. coli* K99, *Cryptosporidium*, and BRV is 1.82%. In the stool samples in which *E. coli* K99 was detected, it was only coinfected with *Cryptosporidium* and BRV, and no other pathogens were detected.

#### Bovine rotavirus

Using the VP6 gene primers of BRV designed by Guo (23) for PCR detection, 51 (23.18%) of 220 stools were positive, of which 16 (7.28%) were infected by BRV alone, and the rest were mixed infection (15.91%). The two highest proportions of mixed infections were BRV and *Cryptosporidium* (7.73%), followed by BRV and *E. coli* K99 (4.09%), and then BRV and BCoV (2.73%).

#### Bovine coronavirus

Using the Nsp10 gene primers in ORF1a of BCoV designed by Guo (23), 26 (11.82%) of 220 stools were positive, of which 8 (3.64%) were infected alone and 18 (8.18%) were infected with mixed infection. The two highest proportions of mixed infections were BCoV and *Cryptosporidium* (4.55%), followed by BCoV and BRV (0.91%), and then BCoV and *E. coli* K99 (0.91%).

#### Bovine kobuvirus

Using the 3D gene primers of BKoV designed by Shi et al. (20) for PCR detection, 7 (3.18%) of 220 stools were positive, all of which were mixed infections. BKoV was predominantly coinfected with *Cryptosporidium* (1.82%) and BRV (1.36%).

#### Bovine astrovirus

PCR detection using the primers of the ORF1a gene of BoAstV reported by Shi (20) showed that 12 (5.46%) of 220 stools were positive, of which 1 was infected alone (0.45%), and the rest were mixed infection (5.00%). The two highest proportions of mixed infections were BoAstV and *Cryptosporidium* (2.73%), followed by BoAstV and BRV (1.36%).

#### Bovine torovirus

The detection was executed using primers designed by Park (22) for the M gene of BToV to PCR. The results showed that 9 of 220 stools (4.09%) were positive, of which 1 was a single infection (0.45%), and the rest were mixed infections (3.64%), suggesting BToV is likely coinfected with two or more pathogens. In a sense, significant diarrhea symptoms only occur when BToV is coinfected with other pathogens.

#### Coccidia

Through the McMaster Egg Slide Counting Chamber, four cases (6.90%) of 58 calves (≥18 days) were detected positive for *coccidia* in this study, including two cases in Wuzhong and two cases in Yinchuan. The OPG levels of the two cases were 4,100 and 8,600 in WuZhong, and the calf ages were 89 and 84 d. The OPG content of the 2 cases was 17,200 and 19,000 in Yinchuan, and the age of the calf was 28 and 27 d. The specific results are shown in Table 3.

**Table 3:** The details of *coccidia* infections in calf diarrhea.

**Cities**	**Number of detected calves**	**Number of infected calves**	**Infection rate (%)**	**OPG (pcs/g)**	
				**Range**	**Average**
Wuzhong	23	2	8.70	4,100–8,600	6,350
Yinchuan	12	2	16.67	17,200–19,000	18,100
Shizuishan	6	0	0	0	0
Zhongwei	8	0	0	0	0
Guyuan	9	0	0	0	0
Total	58	4	6.90	4,100–19,000	12,225

**Figure 2:**
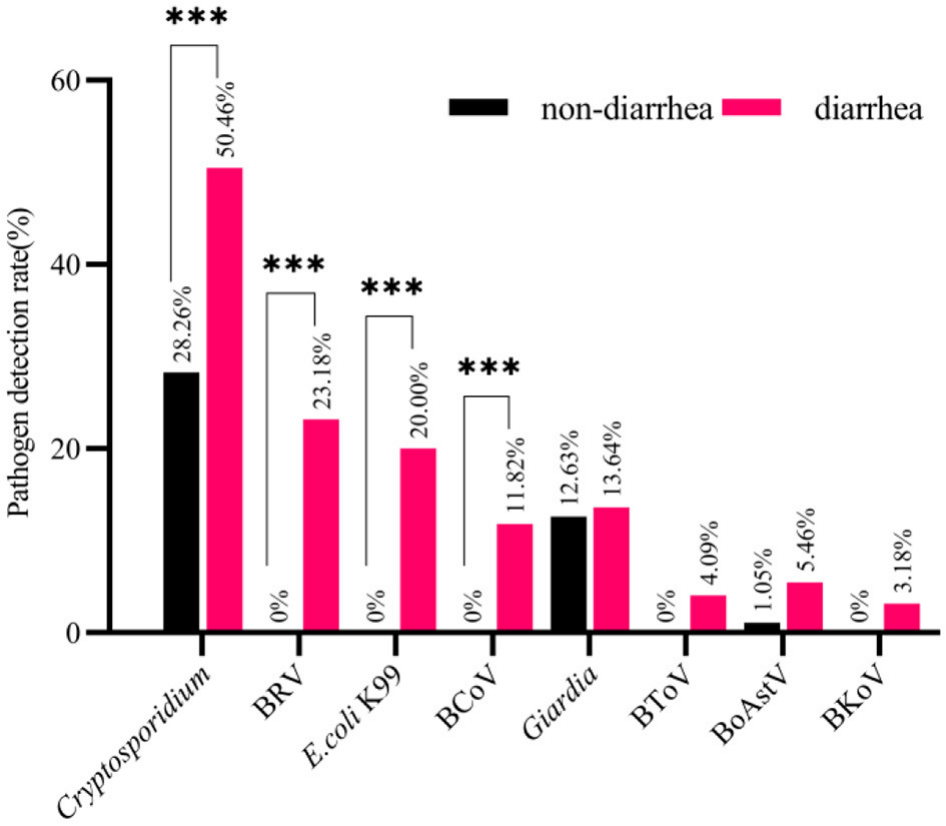
The Pathogens detection rate of diarrhea samples and the Pathogens detection rate of normal samples. The number of pathogen detection and non-detection in normal stool samples with the number of pathogen detection and non-detection in diarrhea stool samples were calculated, and then chi-square test was performed (mean ± standard deviation) ***p < 0.01.

Together, a total of nine pathogens causing diarrhea including bacteria, viruses and parasites were detected in this study. The single infection rate of the detected pathogens is shown in Figure 2. The details of a single infection are shown in Tables 4, 5 for details of a mixed infection.

**Table 4:** The details of calf diarrhea single infection.

**Pathogens**	**Number**	**Percent (%)**
*Cryptosporidium*	53	24.09
BRV	17	7.73
*E. coli* K99	14	6.36
*Giardia*	9	4.09
BCoV	8	3.64
BToV	1	0.46
BoAstV	1	0.46
*Coccidia*	1	0.46
BKoV	0	0
Total	104	47.27

**Table 5:** The details of calf diarrhea mixed infection.

**Pathogens**	**Number**	**Percent (%)**
*E. coli* K99 & *Cryptosporidium*	13	5.91
BRV & *Cryptosporidium*	12	5.45
*E. coli* K99 & BRV	9	4.09
*Cryptosporidium* & *Giardia*	9	4.09
BCoV & *Cryptosporidium*	7	3.18
BoAstV & *Cryptosporidium*	5	2.27
BRV & BCoV	3	1.36
BCoV & *Cryptosporidium* & *Giardia*	2	0.91
BRV & *Cryptosporidium* & *Giardia*	2	0.91
*E. coli* K99 & *Cryptosporidium* & *Giardia*	2	0.91
*E. coli* K99 & BCoV	1	0.45
BToV & BoAstV	1	0.45
BToV & *Giardia*	1	0.45
BoAstV & *Giardia*	1	0.45
BRV & *Giardia*	1	0.45
BToV & *Coccidia*	1	0.45
BKoV & *Cryptosporidium*	1	0.45
BRV & BCoV & BKoV	1	0.45
BRV & BToV & BKoV	1	0.45
BRV & BToV & BoAstV	1	0.45
BCoV & BKoV & *Coccidia*	1	0.45
*E. coli* K99 & BCoV & *Giardia*	1	0.45
*E. coli* K99 & BRV & *Cryptosporidium*	1	0.45
*E. coli* K99 & BoAstV & *Cryptosporidium*	1	0.45
BRV & BCoV& BoAstV& *Giardia*	1	0.45
BRV & BToV& BKoV& *Cryptosporidium*	1	0.45
*E. coli* K99 & BToV & BKoV& *Cryptosporidium*	1	0.45
BCoV & BKoV & *Coccidia* & *Cryptosporidium*	1	0.45
*E. coli* K99 & BRV & BToV & BoAstV & *Giardia*	1	0.45
Total	83	37.73

### Detection rate of different types of pathogens

In this study, 220 stool samples of calves with diarrhea and 95 normal samples of calves were detected. Among bacterial pathogens, *E. coli* K99 and *C. perfringens* were detected, and *Proteus mirabilis* and *Salmonella* were not detected. The primers reported by Jiang (21) were used to identify *C. perfringens*. The *C. perfringens* detected in this study were all type A and had no pathogenicity.

The detection rates of all pathogens from high to low are *Cryptosporidium* (50.46%), BRV (23.18%), *E. coli* K99 (20.00%), BCoV (11.82%), *Giardia* (13.64%), BoAstV (5.46%), BToV (4.09%), BKoV (3.18%). Comparison of detection details between diarrhea stool samples and normal stool samples by chi-square test, among them, the detection rates of *Cryptosporidium* (*p* < 0.01), BRV (*p* < 0.01), *E. coli* K99 (*p* < 0.01), and BCoV (*p* < 0.01) were significantly different between diarrhea stool samples and normal stool samples, while no significant differences were found for the other four pathogens including *Giardia* (*p* = 0.859), BoAstV (*p* = 0.118), BToV (*p* = 0.062), BKoV (*p* = 0.107).

Among the four diarrhea-related pathogens, BRV, *E. coli* K99, and BCoV were not detected in normal stool samples, but *Cryptosporidium* (28.26%) was detected in normal stool samples. The results showed that *Cryptosporidium* had a certain content in normal stool samples and diarrhea stool samples. No clinical diarrhea symptoms in normal stool samples were due to the low content of *Cryptosporidium* in calves. Among other pathogens, *Giardia* (12.63%) and BoAstV (1.05%) were also detected in normal samples and were present in the same situation as *Cryptosporidium*. In contrast, BToV and BKoV were not detected in normal samples, but the results were not significantly different from BToV (4.09%) and BKoV (3.18%) detection rates of diarrhea samples.

#### The detection of pathogen in different cities

A total of 68 stool samples with diarrhea were detected in Yinchuan: *E. coli* K99 was detected in 19 samples (27.94%); BRV in 14 samples (20.59%); BCoV in 6 samples (8.82%); BToV in 2 samples (2.94%); BoAstV in 1 sample (1.47%); BKoV in 2 samples (2.94%); *Coccidia* in 2 samples (2.94%); *Cryptosporidium* in 28 samples (41.18%); *Giardia* in 6 (8.82%) samples.

**Figure 3:**
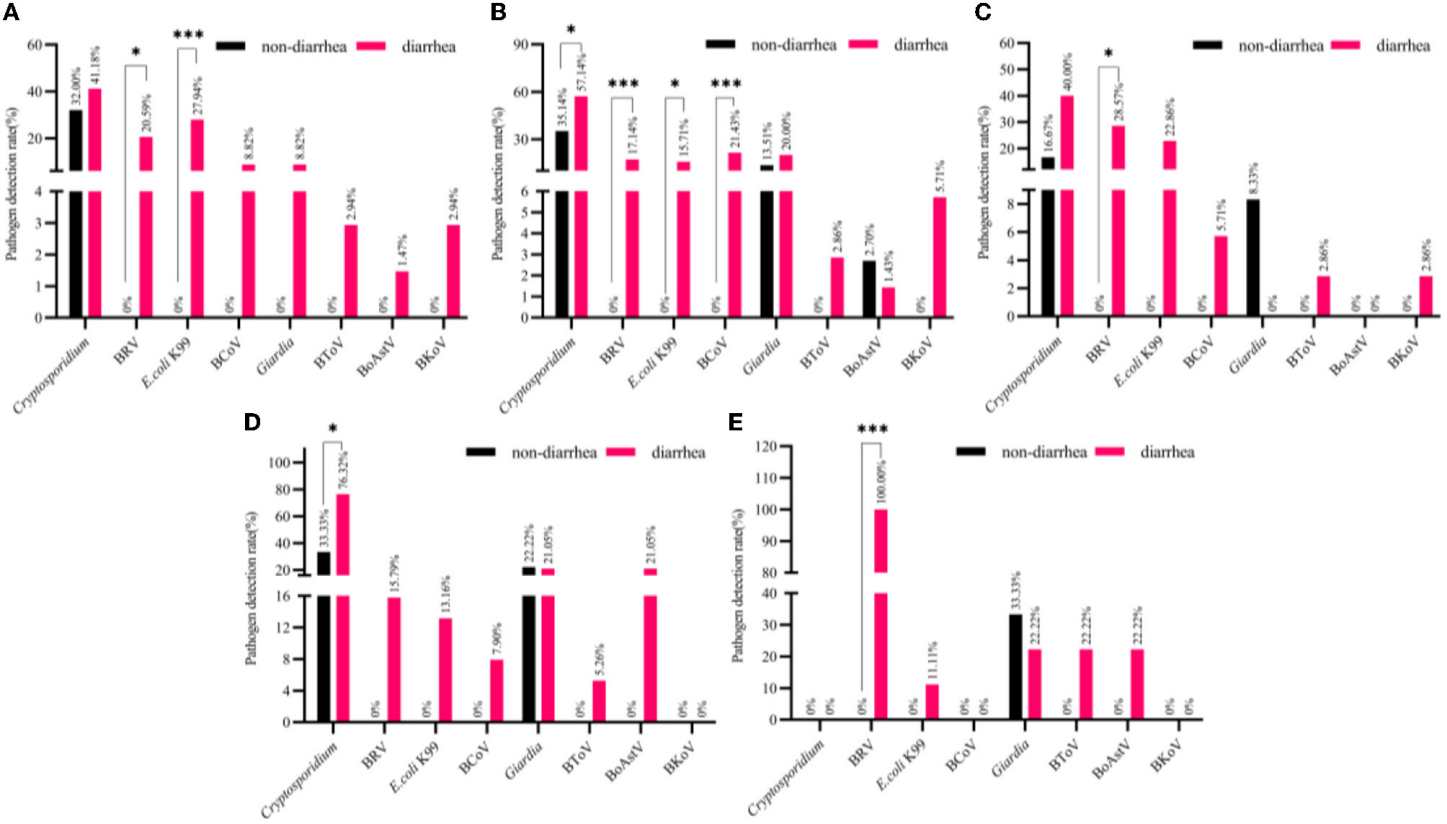
The detection rate of normal and diarrhea samples in different cities [**(A)** Yinchuan, **(B)** Wuzhong, **(C)** Shizuishan, **(D)** Zhongwei, **(E)** Guyuan]. The number of pathogen detection and non-detection in normal stool samples with the number of pathogen detection and non-detection in diarrhea stool samples in different cities were counted for chi-square test (mean ± standard deviation) ***p < 0.01, *p < 0.05.

The Chi-square test showed that the main epidemic cause of diarrhea happened in Yinchuan was *E. coli* K99 (*p* < 0.01), followed by BRV (*p* < 0.05). Although the detection rate of *Cryptosporidium* (41.18%) was the highest in diarrhea stool samples in Yinchuan, it was also the highest in normal stool samples, and the difference was not significant (32.00%). Other pathogens were detected in normal stool samples. The specific results are illustrated in Figure 3A.

Wuzhong detected *E. coli* K99 in 11 (15.71%) of 70 diarrhea stool samples; BRV in 12 (17.14%) samples; BCoV in 15 (21.43%) samples; BToV in 2 (2.86%) samples; BoAstV in 1 (1.43%) sample; BKoV in 4 (5.71%) samples; *Coccidia* in 2 (2.68%) samples; *Cryptosporidium* in 40 (57.14%) samples; *Giardia* in 14 (20.00%) samples.

The Chi-square test showed that the main epidemic pathogen causing diarrhea in Wuzhong calves was BCoV (*p* < 0.01), followed by BRV (*p* < 0.01), *E. coli* K99 (*p* < 0.05), *Cryptosporidium* (*p* < 0.05). Only *Cryptosporidium* was detected in both diarrhea and normal stool samples, and the other three pathogens were not detected in normal stool samples. Although the detection rate of *Giardia* in Wuzhong diarrhea stool samples was higher (20.00%), and it (13.51%) was second only to *Cryptosporidium* (35.14%) in normal stool samples, and the detection rate of *Giardia* in normal stool samples and diarrhea stool samples showed no difference. BToV and BKoV were not detected in normal samples, the detection rates in diarrhea stool samples were low (2.86%, 5.71%), and the difference was not significant. The detection rate of BoAstV in normal stool samples (2.70%) was even higher than that in diarrhea stool samples (1.43%). The results are detailed in Figure 3B.

In Shizuishan, detected 35 diarrhea stool samples including eight samples (22.86%) of *E. coli* K99; 10 samples of BRV (28.57%); two samples of BCoV (5.71%); one sample of BToV (2.86%); one sample of BKoV (2.86%); 14 samples of *Cryptosporidium* (40.00%).

The Chi-square test showed that the main epidemic pathogen causing diarrhea in Shizuishan calves was BRV (*p* < 0.05). Although *Cryptosporidium* (40.00%) and *E. coli* K99 (22.86%) had higher detection rates in diarrhea stool samples, there was no significant difference between them and normal stool samples. In particular, *Giardia* was not detected in diarrhea stool samples, but its detection rate in normal stool samples (8.33%) was second only to *Cryptosporidium* (16.67%). The results are detailed in Figure 3C.

A total of 38 stool samples with diarrhea were detected in Zhongwei: five samples (13.16%) of *E. coli* K99; four samples of BRV (15.79%); three samples of BCoV (7.90%); two samples of BToV (5.26%); eight samples of BoAstV (21.05%); *Cryptosporidium* 29 (76.32%) samples; *Giardia* 8 (21.05%) samples; BKoV and *Coccidia* were not detected.

The Chi-square test showed that the main epidemic pathogen causing diarrhea in Zhongwei calves was *Cryptosporidium* (*p* < 0.05), with a detection rate of 76.32%. The detection rates of other pathogens between diarrhea and normal stool samples were showed no significant difference. The detection rate of *Giardia* in normal stool samples (22.22%) was even higher than that in diarrhea stool samples (21.05%). The results are detailed in Figure 3D.

In Guyuan, a total of nine diarrhea stool samples were detected in *E. coli* K99 in 1 (11.11%); BRV in 9 (100%); BToV in 2 (22.22%); BoAstV in 2 (22.22%); *Giardia* in 2 (22.22%).

The Chi-square test showed that the main epidemic pathogen causing diarrhea in Guyuan calves was BRV (*p* < 0.01). Other pathogens were not significantly different. The detection rate of Giardia in normal stool samples (33.33%) was higher than that in diarrhea stool samples (22.22%), which was similar to the detection of Giardia in Zhongwei and Shizuishan. The results are detailed in Figure 3E.

#### The detection of pathogen in different seasons

The Chi-square test was performed on the number of cattle farms collected in different seasons and the pathogen detection rate of diarrhea fecal samples in each cattle farm. The correlation between the four main pathogens with the significant difference in detection rate in each season and diarrheal calves was analyzed.

**Figure 4:**
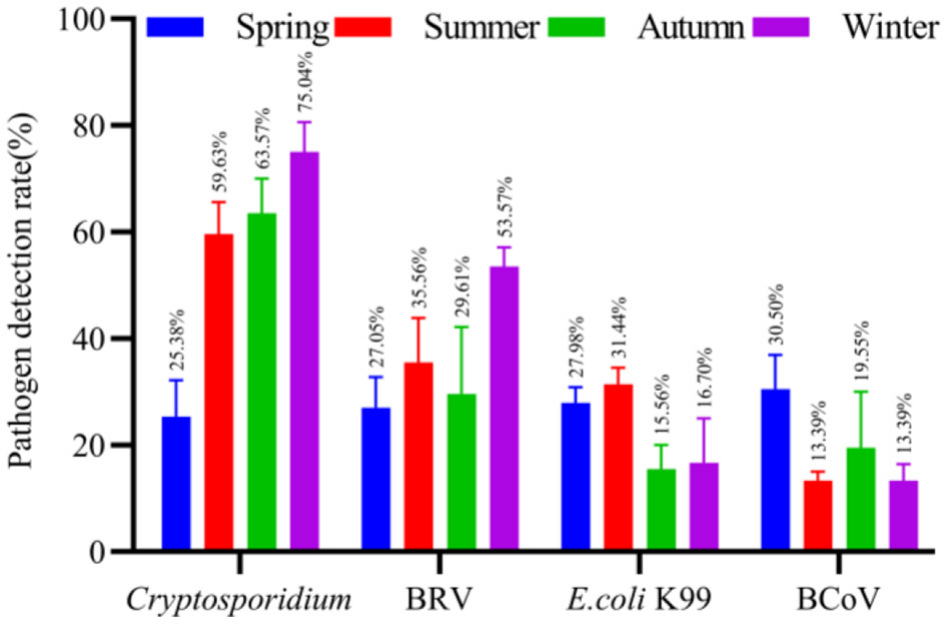
The detection rate of normal and diarrhea samples in different seasons. The number of cattle farms in Ningxia where samples were collected at different seasons, and the detection rates of *Cryptosporidium*, BRV, *E. coli* K99, and BCoV in each cattle farm were counted.

The results showed that the dominant pathogens of diarrhea in spring in Ningxia were BCoV (30.50%), *E. coli* K99 (27.98%), BRV (27.05%) and *Cryptosporidium* (25.38%). In summer, the dominant pathogens of diarrhea were *Cryptosporidium* (59.63%), BRV (35.56%), *E. coli* K99 (31.44%) and BCoV (13.39%). In autumn, the dominant pathogens of diarrhea were *Cryptosporidium* (63.57%), BRV (29.61%), BCoV (19.55%) and *E. coli* K99 (15.56%). In winter, the dominant pathogens of diarrhea were *Cryptosporidium* (75.04%), BRV (53.57%), *E. coli* K99 (16.70%) and BCoV (13.39%). The detail results are illustrated in Figure 4.

After the chi-square test of the entire Ningxia, the pathogens of calf diarrhea that were prevalent in each season have been obtained. Yinchuan and Wuzhong are the concentrated breeding areas of cattle in Ningxia. Analyzing the correlation between the significant pathogens in Yinchuan and Wuzhong in each season and diarrheal calves is more important. Based on the detection rate of different pathogens in the cattle farms of Yinchuan and Wuzhong in different seasons, the average detection rate of pathogens in each cattle farm was calculated, and the epidemic diarrhea pathogens in Yinchuan and Wuzhong in different seasons were obtained.

**Figure 5:**
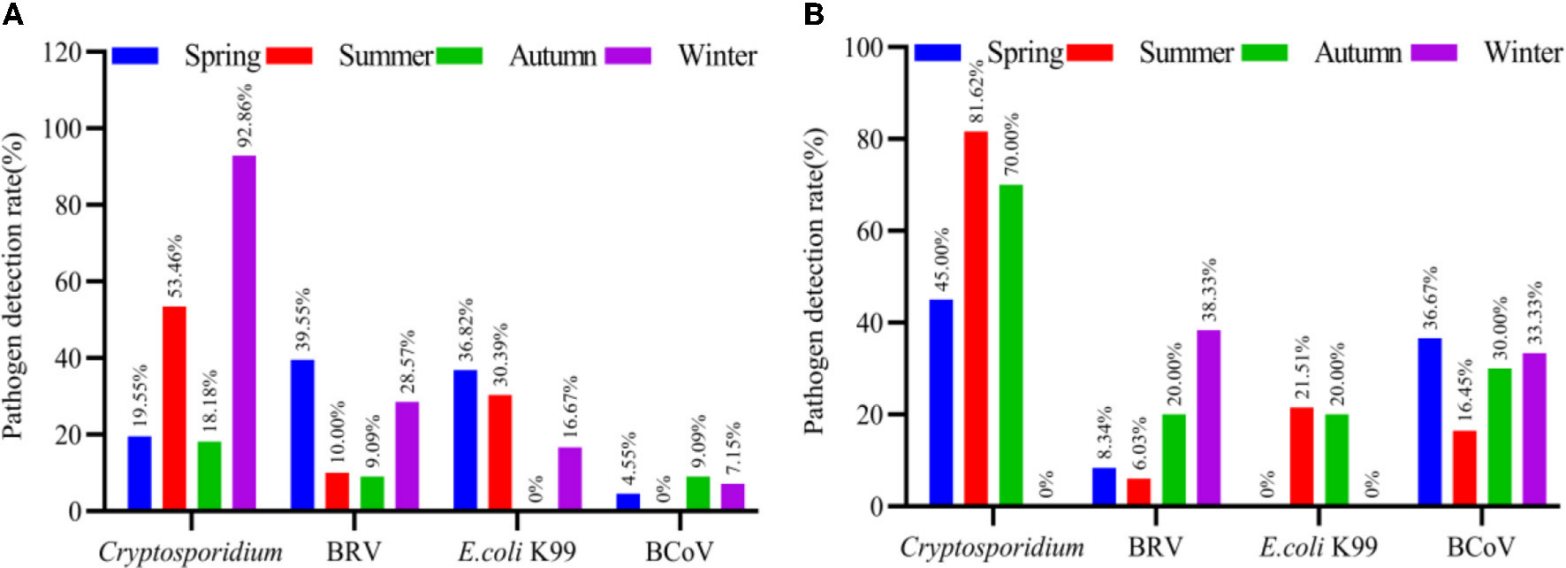
The detection rate of normal and diarrhea samples in different seasons [**(A)** Yinchuan, **(B)** Wuzhong]. The number of cattle farms in Yinchuan and Wuzhong, where samples were collected at different seasons, and the detection rates of *Cryptosporidium*, BRV, *E. coli* K99, and BCoV in each cattle farm were counted.

In Yinchuan, the dominant pathogens of diarrhea in spring were BRV (39.55%), *E. coli* K99 (36.82%), *Cryptosporidium* (19.55%) and BCoV (4.55%). In summer, the dominant diarrhea pathogens were *Cryptosporidium* (53.46%), *E. coli* K99 (30.39%), BRV (10.00%), and BCoV was not detected. In autumn, the dominant diarrhea pathogens were *Cryptosporidium* (18.18%), BRV (9.09%), BCoV (9.09%), and *E. coli* K99 was not detected. In winter, the dominant diarrhea pathogens were *Cryptosporidium* (92.86%), BRV (28.57%), *E. coli* K99 (16.67%), BCoV (7.15%). The results are detailed in Figure 5A.

In Wuzhong, the dominant pathogens of diarrhea in spring were *Cryptosporidium* (45.00%), BCoV (36.67%), BRV (8.34%), and *E. coli* K99 was not detected. In summer, the dominant diarrhea pathogens were *Cryptosporidium* (81.62%), *E. coli* K99 (21.51%), BCoV (16.45%), BRV (6.03%). In autumn, the dominant diarrhea pathogens were *Cryptosporidium* (70.00%), BCoV (30.00%), BRV (20.00%), *E. coli* K99 (20.00%). In winter, the dominant diarrhea pathogens were BRV (38.33%), BCoV (33.33%), *Cryptosporidium* and *E. coli* K99 were not detected. The results are detailed in Figure 5B.

### Distribution of different pathogens in different ages

**Figure 6:**
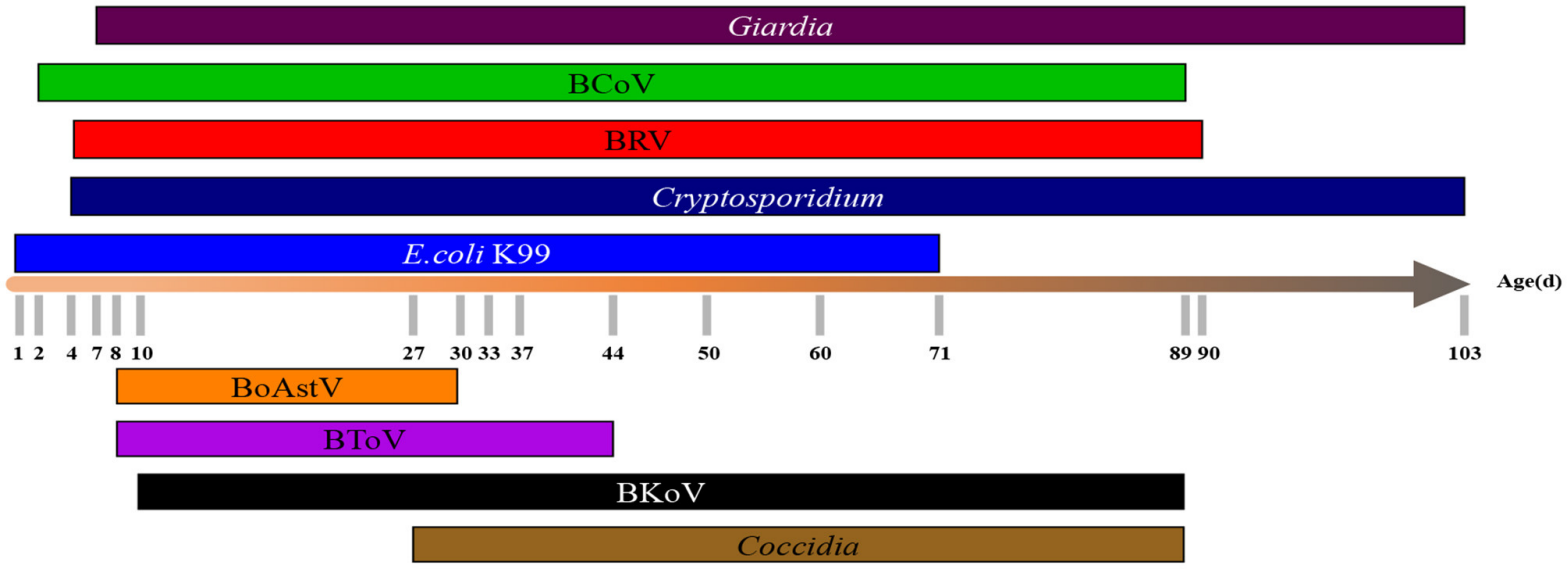
The distribution of different pathogens in different ages. The distribution of 9 pathogens in calves of different ages (1–103 d) was counted.

The earliest onset time and the common age of nine pathogens were illustrated in Figure 6. The earliest onset age of *Cryptosporidium* was 4 days, and the frequent onset age was 5–18 days. The earliest onset age of BRV was 4 days, and the frequent onset age was 7–30 days. The earliest onset age of *E. coli* K99 was 1 day and the common onset age was 8–15 days The earliest onset age of *Giardia* was 7 days, and the most frequent age was 11–30 days. The earliest onset age of BCoV was 2 days, and the most frequent age was 9–26 days. The earliest onset age of BoAstV was 8 days, and the most frequent age was 8-30 days. The earliest onset age of BToV was 8 days, and the most frequent age was 8–44 days. The earliest onset age of BKoV was 10 days, and the most frequent age was 10–26 days. The earliest onset age of *Coccidia* was 27 days.

### Relationship between main diarrhea pathogens and river distribution in Ningxia

Ningxia is a province through which the Yellow River flows, with a length of about 397 km. There are two other tributaries, the Qingshui River and the Kushui River. Among the 23 large-scale cattle farms in this study, 18 cattle farms were close to the river, and the average number of *Cryptosporidium* detected per farm was 7.22, of which 5 cattle farms detected *Cryptosporidium* number ≥10. Among the five cattle farms where no *Cryptosporidium* were detected and where *E. coli* K99, BRV, and BCoV were the main diarrhea pathogens, three cattle farms were not surrounded by a river and one cattle farm was relatively far from a river.

**Figure 7:**
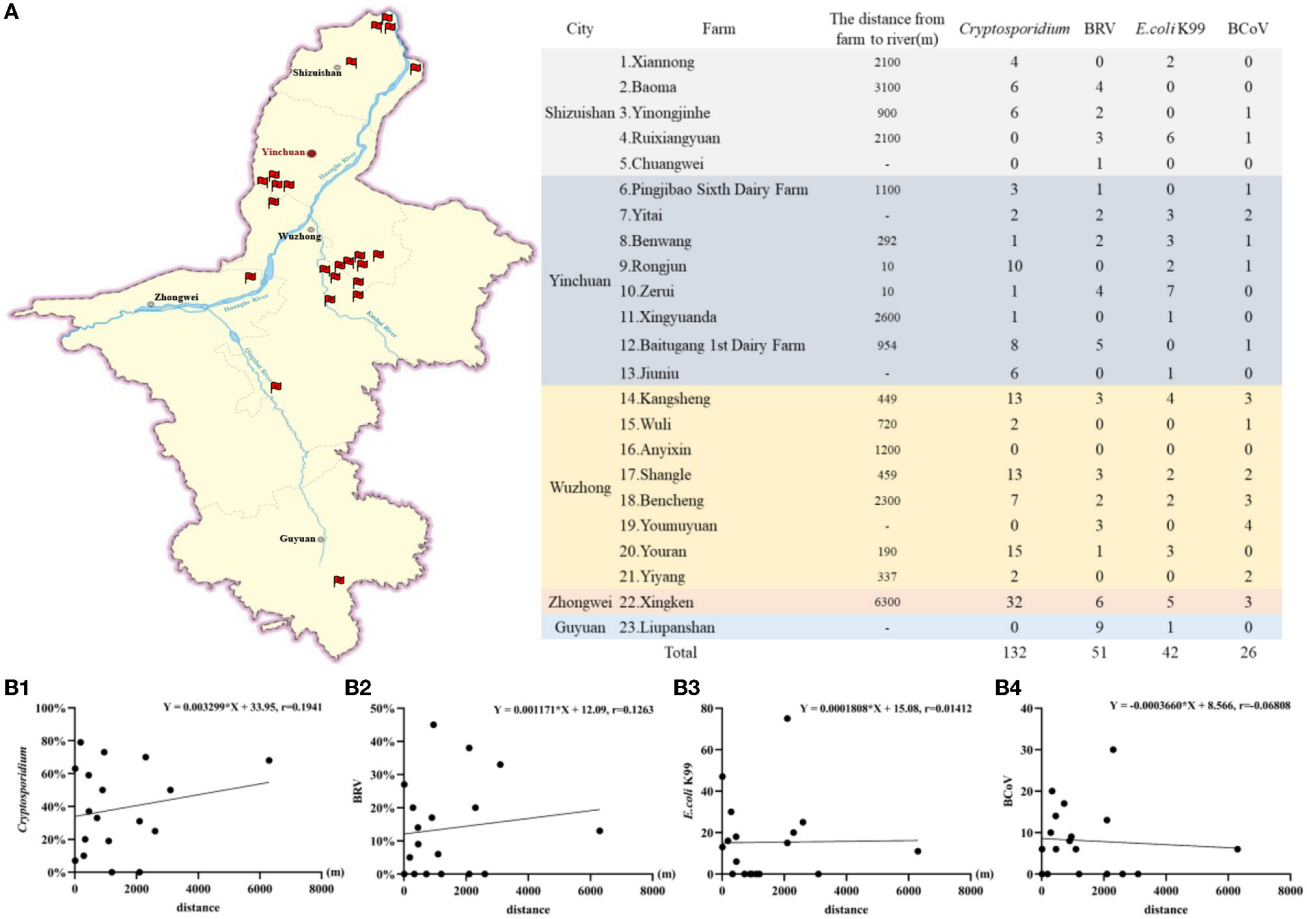
The correlation analysis of main pathogens causing calf diarrhea and river distribution. **(A)** The number of *Cryptosporidium*, BRV, *E. coli* K99, and BCoV detected in cattle farms and distribution of sampling cattle farms and rivers. **(B1)** The correlation between the *Cryptosporidium* detection rate and the distance from cattle farm to river. **(B2)** The correlation between the BRV detection rate and the distance from cattle farm to river. **(B3)** The correlation between the *E.coli* K99 detection rate and the distance from cattle farm to river. **(B4)** The correlation between the BCoV detection rate and the distance from cattle farm to river.

This suggests *Cryptosporidium* is the main diarrhea pathogen in cattle farms, < 500 m from the water source. However, the detection rate of *Cryptosporidium* was positively correlated with the distance from cattle farms to rivers, but not significant (*r* = 0.1941), while the detection rates of *E.coli* K99, BRV, and BCoV were not correlated with the distance from cattle farms to rivers. The specific analysis results are detailed in Figure 7.

## Discussion

Wuzhong is the city with the most types of pathogens and the highest average detection rate, followed by Yinchuan. Because the etiology of calf diarrhea is more complex, in addition to other environmental factors, it is predominantly caused by pathogens [viruses (31), bacteria (1), parasites (32)], especially *Cryptosporidium*, which is principally transmitted by fecal-oral transmission (33). Therefore, calf density is one of the important factors affecting its transmission rate, and Wuzhong, Yinchuan, and some counties in Shizuishan and Zhongwei are the location of cattle breeding areas in Ningxia, and the density of calf herds is extremely high more than other cities. Consistently, like the results reported in other studies, *Cryptosporidium* is an important cause of diarrhea in Ningxia calves (33–35). In this study, a total of 315 stool samples were collected from all five cities in Ningxia, and 137 stool samples (43.49%) were positive for *Cryptosporidium*, including diarrhea samples (50.46%) and normal samples (27.37%). In 2015, researchers reported on *Cryptosporidium* infection in Ningxia and Gansu (35), 150 positive samples (5.09%) were detected in 2,945 stools in both diarrhea and normal calves. The detection rate of our study is significantly higher than 5.09%, which suggests that the infection rate of *Cryptosporidium* in Ningxia is rising year by year. Since December 2011, the detection rate of *Cryptosporidium* in Ningxia has shown a significant increase, from 1.68% (23/1,366) (33) to 50.46% (111/220). The infection of *Cryptosporidium* in calves with diarrhea and normal calves also coexist in this study, which is consistent with the results of the above studies.

At present, the treatment measures for *Cryptosporidium* are only preventive, and there is no effective commercial vaccine on the market to prevent long-term infection in cattle. The increased prevalence is one of the serious problems faced by researchers. Therefore, it is important to take care of deworming cattle in all growth stages and pay attention to biological safety measures.

BRV is the main pathogen that causes calf diarrhea worldwide. It has been also reported in many regions of China. Rotaviruses are a major causative pathogen of diarrhea in humans and animals, involving the deaths of 200,000 children in developing countries and causing economic losses in the livestock industry globally. In this study, the detection rate of BRV in Ningxia from 2021 to 2022 (23.18 %) was lower than the average detection rate of Ningxia over the years (32%), which was lower than the pooled prevalence of BRV in China 46% (6,635/10,677) (36). This is greatly related to the fact that the Ningxia agricultural department pays more attention to the impact of viruses on the cattle industry.

Compared with *Cryptosporidium* and BRV, the infection rates of *E. coli* K99 in Ningxia were relatively low. However, compared with other pathogens, *E. coli* K99 and other pathogenic *Escherichia coli* are still important pathogens causing calf diarrhea. The detection rate of BoAstV in Zhongwei (21.05%) was significantly higher than that in other cities, but it was not the main cause of diarrhea in Zhongwei calves, the reason may be that the BoAstV detected in this study was neurotype rather than diarrhea type. Evolutionary analyses showed that astrovirus strains from bovine brain tissue were closely related to astrovirus strains from humans, pigs, sheep and other animals with neurological symptoms, indicating that cross-species transmission may occur.

To date, *Cryptosporidium, E. coli* K99, BRV and BCoV have been identified as important pathogens prevalent in calf diarrhea in China. In addition, previous studies have demonstrated that BRV can be transmitted to humans directly or through recombination during the evolution of the strain and *Cryptosporidium* and *E. coli* K99, and is typical zoonosis (37). Thus, the in-depth investigation of the above calf diarrhea pathogens is the basis for the prevention and treatment of calf diarrhea, and how to avoid the mixed infection caused by multiple pathogens is of clinical significance. Thus, more efforts should be taken to block the spread of these pathogens in cattle farms and reduce the external factors leading to calf diarrhea. In total, it is possible to reduce the incidence of calf diarrhea.

The area around the reiver is a high-frequency area for parasite reproduction and transmission, and many parasites, including *Cryptosporidium*, can be transmitted through water (38, 39). *Cryptosporidium* in its oocyst stage can remain infectious for many months under cool, moist conditions such as rivers, lakes and ponds (40), and in a relatively dry environment, it is more suitable for the growth of viruses and bacteria (24, 41). The distribution of calf diarrhea pathogens in Ningxia also showed similar characteristics in this study, and how to prevent the spread of the pathogen due to geographic environmental factors is one of the issues the researchers have been facing.

## Conclusion

In this study, *Cryptosporidium* can be detected in both diarrheal calves and normal calves, and other pathogens are a mixed infection of two or more pathogens in the same or different calves. Together, *Cryptosporidium*, BRV, *E. coli* K99 and BCoV are the main pathogens causing calf diarrhea in Ningxia, the remaining four pathogens are mainly infected in the form of mixed infection.

From June 2021 to May 2022, the main pathogens causing calf diarrhea in Yinchuan were *E. coli* K99 and BRV; the main pathogens causing calf diarrhea in Wuzhong are *Cryptosporidium*, BCoV, BRV and *E. coli* K99; BRV was the main pathogen causing calf diarrhea in Shizuishan; *Cryptosporidium* was the main pathogen causing calf diarrhea in Zhongwei; BRV was the main pathogen causing calf diarrhea in Guyuan.

Different seasons had a more obvious effect on the detection rate of calf diarrhea-related pathogens. In addition, the rivers had an effect on the detection rate of *Cryptosporidium*. In conclusion, the distribution of diarrhea pathogens in Ningxia calves is associated with geographical and environmental factors.

## Data availability statement

The original contributions presented in the study are included in the article/supplementary material, further inquiries can be directed to the corresponding authors.

## Ethics statement

The animal study was reviewed and approved by Executive Committee of Laboratory Animal Management and Ethics Inspection of Northwest A&F University, Xianyang, China. Written informed consent was obtained from the owners for the participation of their animals in this study.

## Author contributions

KG, XK, and HG designed the experiments. DW, LZ, and WD carried out the experiments. HG and CL collected samples. DW wrote the manuscript. XZ and YL contributed to data analysis and helped complete the experiments. All authors discussed the results and commented on the manuscript.
